# Supplementary material for: The size of the EB cap determines instantaneous microtubule stability
Source: eLife. 2016 Apr 6;5:e13470. doi: 10.7554/eLife.13470 (PMC4829430; doi:10.7554/eLife.13470)
Supplement: Supplementary file 1. — (B) Fit parameters for the various different threshold models. (C) Correlations between measured parameters. DOI: http://dx.doi.org/10.7554/eLife.13470.019 [file elife-13470-supp1.docx]

SUPPLEMENTAL TABLES

**A. Fit parameters from analysis of comet profiles**

|  | Mal3 Control | 200 nM Mal3 | Tubulin varied |
| --- | --- | --- | --- |
| [Mal3-GFP] (nM) | 0.75 | 200 | 1 |
| *k*_m_ (s^-1^) | 0.129 (0.007)* | 0.161 (0.009)* | 0.133 (0.009)* |
| *x*_c_ (nm)** | 22 | 20 | 27 |

* s.e.m. estimated from the distribution of rate values obtained from fits to the individual comet profiles composing the average; ** *x*_c_ allows for small shifts of the beginning of the profiles with respect to the microtubule end, to account for effects of binding kinetics and/or an additional unresolved maturation step (for details see (*6*))

**B. Fit parameters for the various different threshold models**

| **Threshold model** | **Figure** | **No Mal3** | **200 nM Mal3** | **Tubulin varied** |
| --- | --- | --- | --- | --- |
| End fraction, *f*_crit_ = *n*_crit_ /(13/8nm) | 6A | 0.14 (0.01) | 0.28 (0.03) | 0.20 (0.01) |
| Total number, *N*_crit_ | 6C |  |  | 85 (29) |

s.e.m in brackets.

**C. Correlations between measured parameters**

|  | Delay time, *T* | Mal3 intensity at washout $I_{Mal3\_wo}$ | Shrinkage length, *L*_shrink_ |
| --- | --- | --- | --- |
| Growth speed, *v*_g_ | 0.66 (0.69) | 0.74 | 0.55 (0.51) |
| Mal3 intensity at washout, $I_{Mal3\_wo}$ | 0.78 |  |  |

Correlations are shown for data with 200 nM Mal3-GFP as presented in Figs. 4B, D. E. Correlations for control data without Mal3-GFP as presented in Figs. 2B, E are shown in brackets. All p-values are < 10^-11^.
